# Fetal Tachyarrhythmia - Part II: Treatment

**Published:** 2004-10-01

**Authors:** Martijn A Oudijk, Gerard HA Visser, Erik J Meijboom

**Affiliations:** * Department of Obstetrics and Gynecology, University Medical Center Utrecht,The Netherlands; † Division of Pediatric Cardiology, Central Hospital University of Vaud, Lausanne, Switserland

## Introduction

The decision to initiate pharmacological intervention in case of fetal tachycardia depends on several factors and must be weighed against possible maternal and/or fetal adverse effects inherent to the use of antiarrhythmics.

First, the seriousness of the fetal condition must be recognized. Many studies have shown that in case of fetal tachycardia, there is a significant predisposition to congestive heart failure and subsequent development of fetal hydrops and even sudden cardiac death [1-3]

Secondly, predictors of congestive heart failure have been suggested in several studies, such as the percentage of time that the tachycardia is present, the gestational age at which the tachycardia occurs [4], the ventricular rate [5] and the site of origin of the tachycardia [6]. However, the sensitivity of these predictors is low and they are therefore clinically not very useful. In addition, hemodynamic compromise may occur in less than 24 - 48 hours as has been shown in the fetal lamb [7]  and in tachycardic fetuses [8-9]. On the other hand, spontaneous resolution of the tachycardia has also been described [10].

Thirdly, transplacental management of fetuses with tricuspid regurgitation [11], congestive heart failure or fetal hydrops is difficult [12-13], probably as a result of limited transplacental transfer of the antiarrhythmic drug. In case of fetal hydrops, conversion rates are decreased and time to conversion is increased [13].

Treatment of sustained fetal tachycardia is therefore to be preferred above expectant management, although some centers oppose this regimen and suggest that in cases with (intermittent) fetal SVT not complicated by congestive heart failure or fetal hydrops, conservative management and close surveillance might be a reasonable alternative [16-18].

The most important goal of initiation of treatment is the prevention or resolution of hemodynamic compromise and therefore the prevention of fetal hydrops. In case of treatment the question remains whether to treat prenatally or postnatally, in the latter case after artificial preterm delivery. Several factors play a role in this decision. In case of transplacental treatment, the fetus will be able to thrive in its natural environment and the problems encountered with a preterm delivery will be avoided. One may oppose however, that, with elective preterm delivery and postnatal treatment, monitoring of the infant might be easier. In case of an emergency situation, for instance ventricle fibrillation, physicians may be able to react instantly. But, there is no guarantee of a favourable outcome of this situation in the postnatal setting. This view seems to result in a redirection of the problem in responsibility between different specialties and predisposes the neonate to additional complications of prematurity [19-20]. In addition, an induced preterm delivery, is likely to result in a caesarean section with increased maternal risks and possible effects on subsequent pregnancies [21-23]. The decision as to prenatal or postnatal initiation of treatment mainly depends on gestational age and it is obvious that at an early gestation prenatal treatment is the only reasonable alternative. At term, physicians may differ in opinion whether to treat pre- or postnatally. However, transplacental treatment has proven to be both safe and effective, and serious maternal adverse effects, although theoretically possible, have not been described in literature. It seems therefore logical to treat the fetus in its natural environment through transplacental treatment.

## Direct fetal treatment

Transplacental therapy should be the mode of therapy in nonhydropic fetuses and first choice in hydropic fetuses. However, when conversion to sinus rhythm is not achieved with several maternally administered antiarrhythmic drugs, one may opt for direct fetal therapy.

In the international literature, several modes of administration, intra-umbilical, intra-amniotic, intra-peritoneal, intra-muscular and intra-cardiac, have been described [12,13,24]. The routes of administration all have their specific characteristics. An intra-umbilical injection allows direct access to the fetal circulation and thereby the potential for a quick response to therapy, a characteristic also observed with intra-cardiac injections. However, both of these invasive measures pose a significant risk to the fetus. Intra-peritoneal, intra-amniotic and intra-muscular injections (preferably in the buttock of the fetus), pose less risk to the fetus and provide a more sustained release of the medication.

If one chooses to opt for direct fetal therapy, one must bear in mind that the antiarrhythmic drug will probably distribute to the maternal compartment, unless this compartment is primed with the drug. Therefore, direct fetal therapy should always be administered as an adjunct to maternal administration. In addition, intra-muscular or intra-peritoneal injections that provide a more sustained release into the fetal circulation, as well as an antiarrhythmic drug with a long half-life are to be preferred as this will minimize the number of invasive procedures required.

These ways of administration show a significant mortality, but it is unclear if these deaths are attributable to the invasive nature of the treatment or to the severity of the underlying condition [12-25]. The direct fetal treatment approach should only be used in cases of fetal tachycardia complicated by hydrops with resistance to transplacental multidrug therapy.

## Transplacental treatment

Numerous drugs have been proposed in the international literature for the treatment of fetal tachycardia. The most used and successful drugs are digoxin, sotalol, flecainide and amiodarone. This review article will focus on these drugs.

### Digoxin

Digoxin, a digitalis glycoside, has positive inotropic and negative chronotropic properties, resulting in an increase in cardiac output and a decrease in heart rate. In addition, it prolongs the refractoriness of the AV node. Digoxin has been the drug of first choice in many centers, and is probably the most used drug in the treatment of fetal tachycardia. Reported fetal:maternal(F:M) plasma concentration ratios vary between 0.4 and 0.9 [26-28]. However, in case of fetal hydrops, this ratio is reduced, which may result in failure of treatment. Conversion to sinus rhythm is achieved in approximately 50 % in nonhydropic SVT and of 45% in AF [29-32]. In tachycardia complicated by hydrops, disappointing conversion rates of 15-25 % have been reported [33-34]. It has few adverse effects, and is relatively safe to use. Maternal adverse effects are confined to nausea, vomiting and headache, mostly related to overdosing. Cardiac adverse effects are ventricular extrasystoles and heart block. Digoxin seems to have a relatively low fetal mortality rate.

In conclusion, digoxin is a safe drug in the treatment of fetal tachycardia, however, its use results in relatively low conversion rates, and frequently second line drugs are required to achieve sinus rhythm. Some reports have suggested intravenous administration of digoxin, which may be a more effective approach [35].

### Flecainide

Flecainide, a class IC antiarrhythmic agent, depresses conduction throughout the myocardium and prolongs the refractory period. It has been proposed as an effective drug in the treatment of SVT, especially SVT associated with hydrops, either as drug of first choice or in combination with digoxin. The transplacental transfer is good, with F:M ratios ranging from 0.5 to 0.97 [36-38]. Several studies have provided us with valuable data, reporting excellent conversion rates. It has been used as drug of second choice in nonhydropic SVT, and drug of first choice in hydropic SVT resulting in conversion rates ranging from 75 – 92 % [39-41].

The adverse effects of flecainide are dizziness, headache, visual disturbances, paresthesia, tremors, flushing, nausea and vomiting. The main concern with the use of flecainide is that in the Cardiac Arrhythmia Suppressoin Trial (CAST), a placebo controlled trial in patients with premature beats after myocardial infarction, there was an increased mortality rate in the flecainide group [42]. Therefore it is suggested that its use should be restricted to life-threatening arrhythmias. Its use in fetal tachycardia therefore is mainly concentrated on SVT complicated by hydrops. It has been associated with intra-uterine deaths, however, the occurrence of an intra-uterine death is a well known complication in fetal hydrops, and we can only speculate on the exact relationship with flecainide.

In conclusion, flecainide is a very potent drug in the treatment of SVT, with or without hydrops. Caution is however, required. It should not be used in fetal AF as it may increase the ventricular response.

### Amiodarone

Amiodarone, a class III antiarrhythmic agent, prolongs the repolarization of the myocardium. The drug has received some attention in the past [43-45], but has gained much popularity lately. The transplacental transfer is relatively low, F:M ratio of 0.2 – 0.4 [46]. Data on the use of amiodarone was until recently confined to case reports and some small studies, with success rates of approximately 50 %, bearing in mind that it mostly concerned cases in which other treatment options had failed. Recently, a large study by Strasburger et al. was published in which amiodarone was initiated in drug-refractory fetal tachycardia complicated by hydrops. A high success rate of 93 % in SVT was accomplished, and a lower conversion rate of 33 % in AF [47].

The adverse effects of amiodarone are of possible concern: in some neonates a mild transient biochemical hypothyroidism was detected, which required treatment in only 1 neonate who received amiodarone for a prolonged period after birth. In one mother, treatment had to be stopped because of the development of a photosensitive skin rash and thrombocytopenia. No intra-uterine deaths occurred in this study.

In conclusion, amiodarone is a very successful drug, and suitable as second line treatment, especially in hydropic SVT.

### Sotalol

Sotalol is a beta blocking agent with additional class III antiarrhythmic properties. Several studies in the past years have shown its efficacy, and it has been encorporated in many treatment protocols.

The transplacental transfer is excellent with a F:M ratio of 1:1 [48]. The success rate of sotalol as a single therapy in the treatment of atrial flutter in the reported studies was approximately 65 %, and reached 80 % after the addition of digoxin [49]. This compares favorably with other reports [32]  and it is concluded that sotalol is a superior drug in the treatment of AF, both nonhydropic as hydropic. The success rate in fetal SVT was approximately 55 % with sotalol as a single drug and reached 75 % after the addition of digoxin. These results are comparable with other proposed treatment protocols. Maternal adverse effects are not frequent, and mostly related to its ß blocking properties. However, sotalol has been associated with intra-uterine deaths, mainly in hydropic cases with SVT [50]. Ventricle fibrillation might have been the cause of death and therefore, proarrhythmic effects of sotalol are of concern.

In conclusion, sotalol is a very potent drug in the treatment of fetal AF, with or without hydrops, and is recommended as drug of first choice. In SVT, caution is required and the risk of proarrhythmia should be minimized, low initiation dosages and stepwise dosage increases are recommended. In addition, close monitoring, especially during the initiation phase is recommended. Sotalol seems contraindicated in SVT complicated by hydrops.

## Conclusions

Fetal tachycardia is a serious condition in which the fetus is at significant risk for fetal hydrops, neurological damage and intra uterine death. In cases with intermittent tachycardia and no signs of hemodynamical compromise, one may opt for expectant management (close monitoring is mandatory), however, in case of sustained tachycardia we strongly recommend to initiate transplacental treatment. Treatment of fetal tachycardia should be executed in experienced maternal-fetal medicine centers and close monitoring of fetal well-being by ultrasound, especially in the initiation phase, is recommended. Before initiation of therapy, pre-existing maternal arrhythmias and/or a prolonged QT segment should be excluded by a thorough examination of medical history and a maternal ECG. It is wise to have the maternal ECG repeated during treatment, especially in case of dosage increase, or the addition of second-line treatment.

On the basis of the international literature and personal experience we have developed several treatment protocols. In case of fetal AF, either hydropic or nonhydropic, we advocate sotalol as drug of first choice, and digoxin as drug of second-choice (**Utrecht protocol 1**). In case of fetal SVT without hydrops, we recommend digoxin as drug of first choice and sotalol as drug of second choice (**Utrecht protocol 2**). In case of fetal SVT complicated by hydrops two options are available, either flecainide as drug of first choice and digoxin as second choice, or digoxin intravenous (maternal), followed by amiodarone as drug of second choice (**Protocol 3**).

## Utrecht Protocol 1: Fetal Atrial Flutter

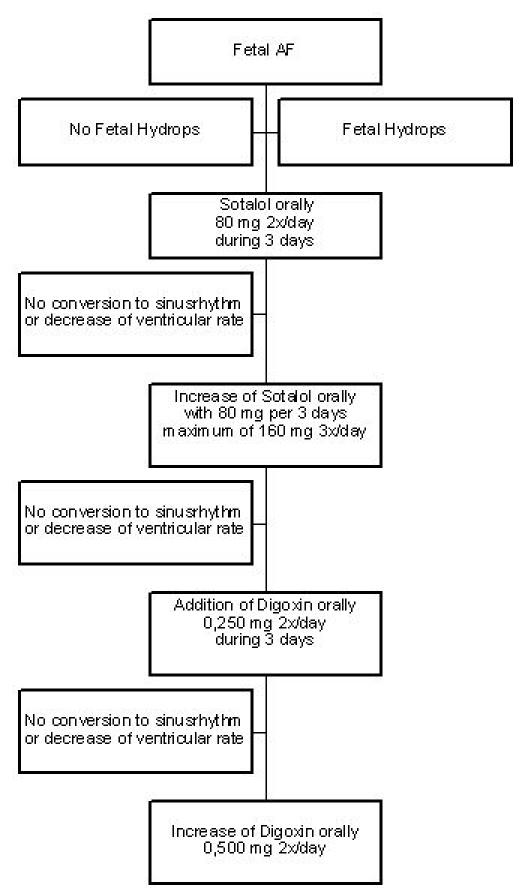



## Utrecht Protocol 2: Fetal Nonhydropic SVT

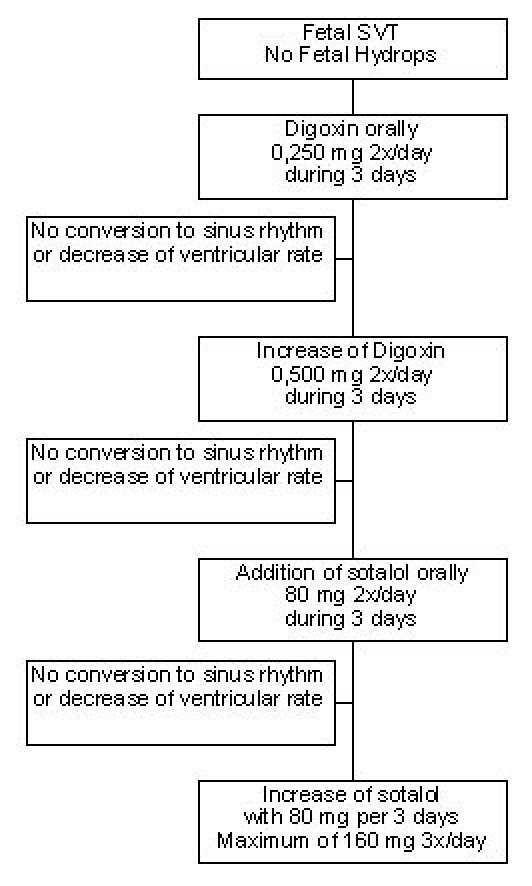



## Protocol 3: Fetal Hydropic SVT


